# Low Dose Radiation Therapy Induces Long-Lasting Reduction of Pain and Immune Modulations in the Peripheral Blood – Interim Analysis of the IMMO-LDRT01 Trial

**DOI:** 10.3389/fimmu.2021.740742

**Published:** 2021-10-12

**Authors:** Anna-Jasmina Donaubauer, Ina Becker, Thomas Weissmann, Birgitta M. Fröhlich, Luis E. Muñoz, Thomas Gryc, Manuel Denzler, Oliver J. Ott, Rainer Fietkau, Udo S. Gaipl, Benjamin Frey

**Affiliations:** ^1^ Translational Radiobiology, Department of Radiation Oncology, Universitätsklinikum Erlangen, Friedrich-Alexander-Universität Erlangen-Nürnberg (FAU), Erlangen, Germany; ^2^ Department of Radiation Oncology, Universitätsklinikum Erlangen, Friedrich-Alexander-Universität Erlangen-Nürnberg (FAU), Erlangen, Germany; ^3^ Department of Internal Medicine 3 - Rheumatology and Immunology, Friedrich-Alexander-University Erlangen-Nürnberg (FAU), Universitätsklinikum Erlangen, Erlangen, Germany

**Keywords:** low dose radiation therapy (LDRT), immune status, immunophenotyping, chronic degenerative and inflammatory diseases, subjective pain level, x-rays

## Abstract

The treatment of chronic inflammatory and degenerative diseases by low dose radiation therapy (LDRT) is promising especially for patients who were refractory for classical therapies. LDRT aims to reduce pain of patients and to increase their mobility. Although LDRT has been applied since the late 19th century, the immunological mechanisms remain elusive. Within the prospective IMMO-LDRT01 trial (NCT02653079) the effects of LDRT on the peripheral blood immune status, as well as on pain and life quality of patients have been analyzed. Blood is taken before and after every serial irradiation with a single dose per fraction of 0.5Gy, as well as during follow-up appointments in order to determine a detailed longitudinal immune status by multicolor flow cytometry. Here, we report the results of an interim analysis of 125 patients, representing half the number of patients to be recruited. LDRT significantly improved patients’ pain levels and induced distinct systemic immune modulations. While the total number of leukocytes remained unchanged in the peripheral blood, LDRT induced a slight reduction of eosinophils, basophils and plasmacytoid dendritic cells and an increase of B cells. Furthermore, activated immune cells were decreased following LDRT. Especially cells of the monocytic lineage correlated to LDRT-induced improvements of clinical symptoms, qualifying these immune cells as predictive biomarkers for the therapeutic success. We conclude that LDRT improves pain of the patients by inducing systemic immune modulations and that immune biomarkers could be defined for prediction by improved patient stratification in the future.

## Introduction

Chronic degenerative and inflammatory diseases, such as osteoarthritis have an increasing prevalence in western countries over the last decades. As the prevalence of these diseases is closely connected to rising mean age in the population, incidence will further increase in the future (1). The term chronic degenerative and inflammatory diseases covers a broad spectrum of conditions ranging from rheumatoid arthritis, which is characterized by steadily progressing inflammatory processes in multiple joints (2), to conditions that are characterized mainly by local, degenerative processes in bone or cartilage tissue, such as the elbow syndrome or osteoarthritis (1). In detail, rheumatoid arthritis is considered as a progressing autoimmune disease that is caused by genetic, as well as environmental factors, such as nutrition, smoking or hormones (3). During the course of the disease, patients experience a severe chronic inflammation of several synovial joints that is followed by a destruction of cartilage and bone (2). For other chronic degenerative diseases on the other hand, such as osteoarthritis, typical risk factors are a high age, obesity or chronic mechanical overload of the joint (4). Those factors lead to a slow degradation of the joint’s cartilage and finally even of the bone tissue and a subsequent deformation of the affected joint. In line with inflammatory diseases, an activation of the immune system and inflammatory processes are also a symptom of chronic degenerative diseases in very advanced stages (5). A further common symptom of chronic degenerative and inflammatory diseases is intense pain in the affected joints and a resulting reduction of the joint’s mobility that affects patient’s quality of life (6).

As chronic degenerative and inflammatory diseases are heterogeneous, the possible treatment options are very broad and depend on the specific diagnosis and stage of disease. As those diseases are progressive, the therapeutic intervention primarily aims to decelerate the destructive and inflammatory processes and to reduce the pain. Depending on the diagnosis, patients can profit from non-pharmacological treatment strategies, such as orthoses, physiotherapy or a weight reduction. Nonetheless, these options often fail to induce a sufficient pain relief. Therefore, pharmacological interventions with non-steroidal anti-inflammatory drugs (NSAIDs), disease-modifying anti-rheumatic drugs (DMARDs), injections or even surgical interventions are considered in advanced stages (6). Even though, there is a plethora of therapeutic options, about 25% of all patients do not respond sufficiently to those interventions or lose their responsiveness over time (7).

Especially for those patients with insufficient therapeutic responses, low dose radiation therapy (LDRT) is a promising alternate therapy. For over 100 years, LDRT is well-known for the pain relieving effects in joint diseases that can last at least for up to one year (8). LDRT is usually applied as a serial, local irradiation with X-Rays, delivered on the affected joint only. The single dose ranges between 0.5 and 1.0 Gray (Gy) and the total dose does usually not exceed six Gy (9, 10). Radiation-induced side effects do play a minor role in LDRT (11).

Even though the analgesic effects of LDRT are well known, the molecular mechanisms are not yet characterized in detail. As pain is often closely connected to inflammatory processes, a modulation of the inflammatory reaction is very likely a central mechanism of action of LDRT. Indeed, numerous preclinical studies focus on the immunological modes of action of LDRT. In general, it was found that LDRT downregulates preexisting inflammatory and bone degrading processes. Up to today, various immune-modulating mechanisms have been found in preclinical models that support this hypothesis. In leukocytes, LDRT increases the apoptosis rate and reduces the adhesion of the immune cells to the endothelium. Moreover, the secretion of pro-inflammatory cytokines such as IL-6 and TNF-α, as well as chemokines is downregulated and the secretion of anti-inflammatory cytokines, such as TGF-β is enhanced. Especially macrophages seem to be modulated by LDRT, as they reduce the secretion of reactive oxygen species and enhance the clearance of apoptotic cells. In summary, an anti-inflammatory microenvironment is formed by LDRT in the affected tissue (12–16). In line with those immunological alterations, LDRT also impacts positively on the bone metabolism, as bone degradation is decelerated and the formation of new bone is enhanced and therefore degenerative processes are ameliorated (17).

All of the immune-modulating effects described above have been examined in preclinical models or *in vitro* studies. On the other hand, a plethora of patient studies are being conducted on the effectiveness of LDRT (18). However, those studies focus primarily on clinical parameters and the analgesic effects (19, 20), rather than unraveling the underlying immunological modes of action.

In order to prospectively examine the immunological mechanisms of LDRT for the first time in a clinical trial, the IMMO-LDRT01 study was initiated in 2015. In this prospective, observatory trial, patients suffering from various chronic degenerative and inflammatory diseases have been enrolled. During the course of the study, these patients are examined for pain-related factors, but additionally a detailed immune status of the peripheral blood is analyzed. Peripheral blood can be drawn easily and be repeated without an additional risk for the patients compared with repeated biopsies tissue and thereby allows a detailed and longitudinal immune monitoring for future definition of prognostic and predictive signatures (21). Thereby, the immunological modulations of LDRT before and after the therapy has been determined for the first time *in vivo* in patients. Here we present an interims analysis with 125 of 250 patients of the IMMO-LDRT01 trial. Our findings prove that LDRT does not only significantly improve clinical symptoms and pain, but also modulates peripheral immune cell numbers, as well as the activation state of distinct immune cells. Certain modulations correlate to the therapeutic outcome and might therefore be valuable in the future for defining predictive and prognostic biomarkers for LDRT.

## Material and Methods

### Study Design

The IMMO-LDRT01 trial (NCT02653079) was initiated in 2015. This prospective, observatory study aims to unravel the immunological mechanisms underlying LDRT, as well as proving the pain-relieving effects. Informed consent was obtained from all subjects involved in the study.

**Figure 1:**
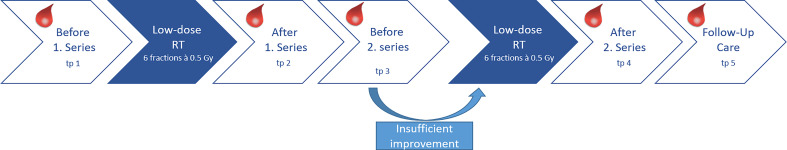
Study design of the prospective IMMO-LDRT01 trial. Examinations are performed before and after the first series of irradiation (tp 1 and 2), before and after the second series of irradiation (tp 3 and 4), as well as for the follow-up appointment three months later (tp 5). The blood drops indicate the time points of the blood withdrawals. Patients fill out questionnaires on life quality and pain levels at the same time points.

After enrollment, patients undergo a serial irradiation with six fractions of a single dose per fraction of 0.5 Gy delivered over three weeks with at least 48 hours between the fractions. If the patient is not subjectively satisfied with the therapeutic outcome, a second radiation series is applied three months later according to the same scheme as before. Radiation therapy is applied using an orthovoltage technique. In detail, an X-ray device (from Xstrahl LTD, Suwanee, GA, US) was used with 180kV and 10mA, a 0.2 mm Cu filter and a focus skin distance of 50 cm. Three months after the completion of LDRT a follow-up visit is performed. **Figure 1** depicts the study design of the trial.

As immunological modulations by LDRT are examined in this trial, a detailed immune status was analyzed from peripheral blood. Therefore, patients underwent blood withdrawals at indicated time points before and after every irradiation series, as well as for the follow-up appointment (**Figure 1**). In addition, the patients filled out a detailed questionnaire on their pain level, health status and quality of life at the same time points. In detail, the first examination time point was on the day the first radiation series started and the second time point was on the day of the last radiotherapy session of the first series (three weeks later). Time point 3 was three months later on the day the second series started and time point 4 was again three weeks later when the last fraction was applied. The final examination was performed after additional three months, when the patients received their follow-up care.

The study was conducted according to the guidelines of the Declaration of Helsinki, and approved by the Institutional Review Board of the Friedrich-Alexander Universität Erlangen-Nürnberg (protocol code: 289_15B; date of approval: 12.11.2015).

Informed consent was obtained from all subjects involved in the study. Written informed consent has been obtained from the patients to publish this paper.

### Patient Cohort

For this planned interim analyses, 125 out of planned 250 patients suffering from chronic degenerative and inflammatory diseases have been enrolled. All patients received alternative treatments before being referred to the Department of Radiation Oncology of the Universitätsklinikum Erlangen for LDRT. The detailed patient characteristics are listed in **Table 1**.

**Table 1:** Patient characteristics.

Factor	Category	n
Total number		125
Age at start	Mean	58
	Range	41-83
Gender	Male	32 (25.6%)
	Female	93 (74.4%)
BMI	Normal (≥ 25)	28 (22.4%)
	Overweight (25-30)	35 (28%)
	Obese (≤ 30)	42 (33.6%)
	N/A	20 (16%)
Number of series	1 series	32 (25.6%)
	2 series	77 (61.6%)
	Drop-out	16 (12.8%)
Indication	Calcaneodynia	45 (36%)
	Osteoarthritis	16 (12.8%)
	Arthritis	10 (8%)
	Elbow syndrome	12 (9.6%)
	Shoulder syndrome	6 (4.8%)
	Achillodynia	9 (7.2%)
	Multiple indications	4 (3.2%)
	Other	23 (18.4%)

### Immunophenotyping of Peripheral Blood

**Figure 2:**
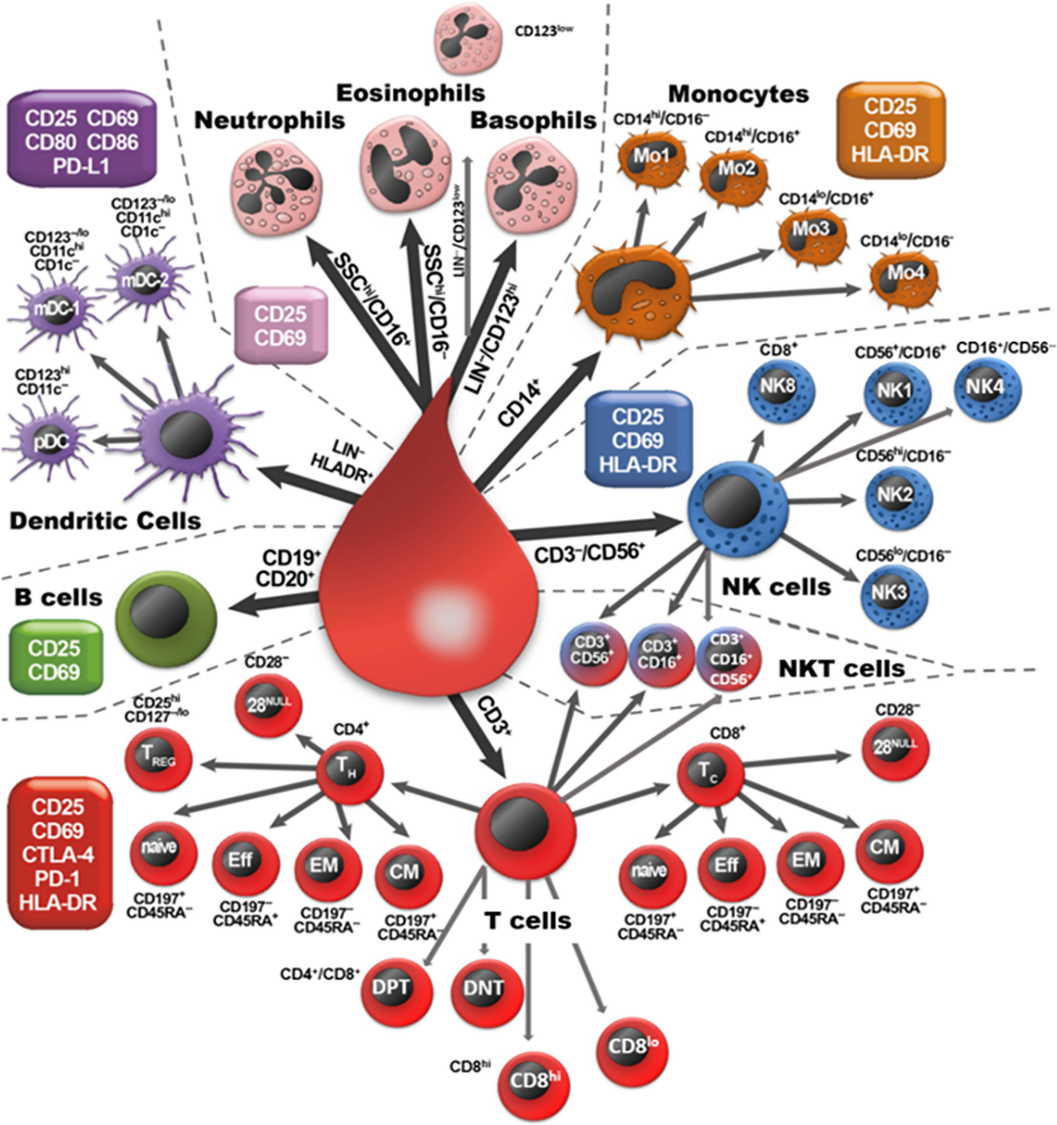
Overview of the analyzed immune cell subpopulations. The scheme presents all nine main immune cell types (neutrophilic, eosinophilic and basophilic granulocytes, monocytes, NK cells, NKT cells, T cells, B cells and DCs) that were identified by the expression of their specific pan marker (big arrows). Those main immune cells were subdivided by analyzing the expression of further surface markers (next to each cell). Finally, all cells are analyzed for the expression of specific activation markers (CD25, CD69, CD80, CD86, PD-L1, HLA-DR, CTLA-4, PD-1), as indicated in the boxes.

In order to determine a detailed immune status from peripheral blood, immunophenotyping was performed. Therefore, whole blood samples were analyzed within three hours after the blood withdrawal by multi-color flow cytometry. Immunophenotyping (IPT) was executed as described in our previously published IPT protocols (22–24). **Figure 2** depicts the gating strategy for the IPT used in this study with all the determined immune cell subtypes and the respective surface molecules that are measured for their identification. A Gallios flow cytometer (Beckman Coulter) in the standard filter configuration was used for data acquisition. The Kaluza Flow Analysis Software (Beckman Coulter), as well as Microsoft Excel were used to calculate immune cell numbers.

### Determination of Pain and Quality of Life

In order to quantify the pain-relieving effects of LDRT patients filled out a detailed questionnaire on their pain level in different daily situations and times, health status, as well as their quality of life. The pain level (and the morning stiffness) were scored on a visual analog scale (VAS) ranging from 10 = worst pain imaginable to 0 = no pain. As most patients experienced a pain reduction that lasted until the follow-up appointment, patients were asked to score their pain level again retrospectively at least six months after the follow-up appointment, in order to determine long-lasting therapeutic effects.

### Statistical Analysis

Microsoft Excel was used for data management and GraphPad Prism 9 and IBM SPSS Statistics 24 were used for the statistical analysis.

The Wilcoxon test was applied to evaluate the differences in the scoring of the retrospective pain level, the absolute immune cell numbers and the activation status of the immune cells. A p-value of < 0.05 was considered statistically significant.

**Figure 3:**
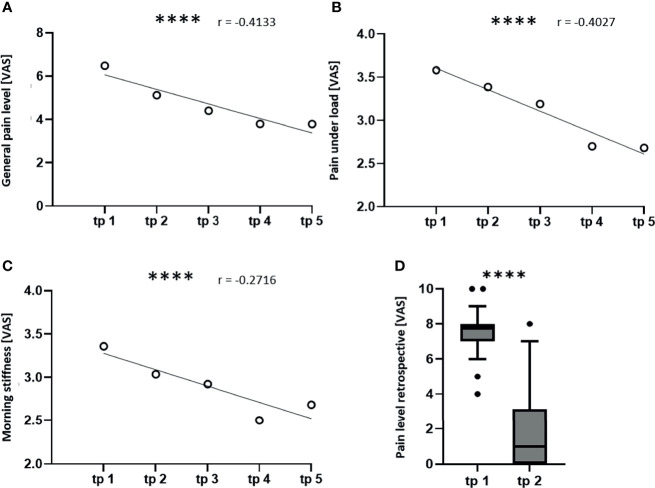
LDRT induces a significant pain reduction. The patients scored their pain level on a visual analogue scale (VAS) ranging from 0 (no pain) to 10 (worst pain imaginable) at the indicated time points (tp). A Spearman correlation and a simple linear regression were performed in order to correlate the pain levels of all patients to the time points **(A–C)**. For the graphical illustration, the mean values of the pain levels were calculated for all five tp. **(A)** shows the correlation of the mean general pain level, **(B)** shows the correlation of the pain under load and **(C)** shows the correlation of the morning stiffness to the study tp (n = 100; ****p < 0.0001). **(D)** shows a box plot for the retrospective determination of the pain level before therapy and at least six months after therapy. A Wilcoxon test was used for the statistical analysis in graph **(D)** (n = 125; ****p < 0.0001).

A Spearman’s rank order correlation was performed to analyze the relationship between the pain level and the study time points, as well as the relationship between the immune cell numbers and the pain level. A p-value of < 0.05 was considered as statistically significant. Afterwards, a simple linear regression was performed. For the graphical illustration the mean values of the pain levels (**Figure 3**) and the immune cell numbers (**Figure 6**) were plotted.

## Results

### LDRT Induces Long-Lasting Analgesic Effects

In order to prove and quantify the pain-relieving effects of LDRT, the patients filled out a questionnaire on their subjective pain level, as well as their health status and quality of life. A more detailed understanding of the pain level was generated by asking for the quality of pain or the pain level in different situations, such as the pain at rest, the pain at night or the morning stiffness. Indeed, differences in the pain level in these different situations were detected. The most prominent findings on the pain level are summarized in **Figure 3**.

The general pain level (**Figure 3A**) shows a strong negative correlation (r = -0.4133) to the study time points. This means that the pain level is reduced during the course of the study from average 6.5 to 3.8 points on VAS after the second series of irradiation (tp 4). Afterwards the pain level stagnates at the time point of the follow-up appointment (tp 5). In addition, the pain level under load (B) is also correlating negatively (r = -0.4027) to the study time points. Here, a reduction of the pain from average 3.6 to 2.7 points on VAS at time point four was observed. Again, the pain level stagnates at the follow-up appointment. Further, the morning stiffness correlates negatively (r = -0.2716) to the time points, showing a reduction from average 3.4 to 2.5 points on VAS with a minimum pain level after the second irradiation series. The detailed statistical parameters, such as the mean, the SD, the SEM and the 95% CI are depicted in **Table 2**. Finally, also the retrospective scoring of the pain level proves that LDRT induces a long-lasting analgesic effect, as the patients report a significant pain reduction even six months after the last irradiation series from about 8 to 1 point on VAS. This indicates that patients score their initial pain level even higher retrospectively. Also, they report a further reduction of their symptoms as they score their current pain level at least six months after therapy with around 1 point on VAS.

**Table 2:** Statistical parameters for the pain levels.

Pain parameter	Time point	Mean	SD	SEM	95% CI
General pain level	tp 1	6,8	2,0	0,3	± 0,5
General pain level	tp 2	5,1	2,1	0,3	± 0,5
General pain level	tp 3	4,4	2,3	0,4	± 0,7
General pain level	tp 4	3,8	2,6	0,4	± 0,8
General pain level	tp 5	3,8	2,6	0,5	± 1,0
Pain under load	tp 1	3,6	0,6	0,1	± 0,2
Pain under load	tp 2	3,4	0,7	0,1	± 0,2
Pain under load	tp 3	3,2	0,7	0,1	± 0,2
Pain under load	tp 4	2,7	0,8	0,1	± 0,3
Pain under load	tp 5	2,7	1,0	0,2	± 0,4
Morning stiffness	tp 1	3,4	1,0	0,1	± 0,3
Morning stiffness	tp 2	3,0	1,0	0,1	± 0,3
Morning stiffness	tp 3	2,9	1,1	0,2	± 0,3
Morning stiffness	tp 4	2,5	1,1	0,2	± 0,3
Morning stiffness	tp 5	2,7	1,2	0,2	± 0,5

### LDRT Modulates Numbers of Distinct Immune Cells of the Peripheral Blood

**Figure 4:**
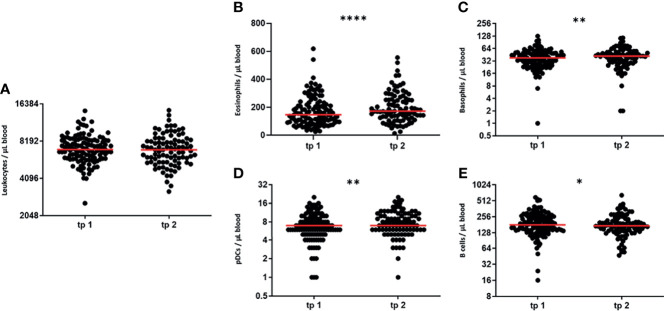
LDRT modulates numbers of certain peripheral immune cells. In order to determine peripheral blood immune cell numbers, immunophenotyping was performed with patient’s blood. Blood withdrawals were performed before (tp 1) and after serial irradiation (tp 2). The absolute immune cell numbers were determined by flow cytometry. The Wilcoxon test was used for the statistical analysis. In the figure the immune cell numbers of the blood leukocytes **(A)**, the eosinophilic and basophilic granulocytes **(B, C)**, the plasmacytoid dendritic cells **(D)** and the B cells **(E)** are presented. (n = 125; *p < 0.05; **p < 0.01 ****p < 0.0001).

A detailed immunophenotyping of the peripheral blood was performed before and after every serial irradiation, as well as for the follow-up appointment (see also **Figure 1**). As analyses of modulations of cellular immune components of the peripheral blood have never been performed in clinical trials with patients receiving LDRT, we first examined which immune cell populations are modulated by a serial irradiation with a total dose of 3 Gy (**Figure 4**).

First, we detected the number of leukocytes remained stable after the first series of irradiation (**Figure 4A**). Nonetheless, we detected slight, but significant modulations in the numbers of 4 out of all examined key immune cell subtypes. Cells of the innate immune system, namely eosinophilic and basophilic granulocytes and plasmacytoid dendritic cells are significantly upregulated after the first serial irradiation (**Figure 4B–D**). Cells of the adaptive immune system on the other hand, namely the B cells, display a slight, but significant decrease of their cell numbers after irradiation (**Figure 4E**). The detailed parameters (Mean, SD, SEM and 95% CI) are depicted in **Table 3**.

**Table 3:** Statistical parameters for the modulation of the immune cells & activation markers.

Immune cell type	Time point	Mean	SD	SEM	95% CI
Leukocytes	tp 1	7258	1849	168,8	± 330,8
Leukocytes	tp 2	7217	2117	219,5	± 430,2
Eosinophils	tp 1	171	105	9,6	± 18,8
Eosinophils	tp 2	200	108	11,2	± 22,0
Basophils	tp 1	41	20	1,8	± 3,6
Basophils	tp 2	44	21	2,2	± 4,3
pDCs	tp 1	7	4	0,3	± 0,6
pDCs	tp 2	8	4	0,4	± 0,8
B cells	tp 1	204	101	9,3	± 18,2
B cells	tp 2	191	95	9,9	± 19,4
CD25+ monocytes	tp 1	2,4	1,9	0,2	± 0,4
CD25+ monocytes	tp 2	1,8	1,1	0,1	± 0,2
HLA-DR+ monocytes	tp 1	86,0	12,0	1,1	± 2,2
HLA-DR monocytes	tp 2	84,0	11,0	1,1	± 2,2
PD1+ TH cells	tp 1	15,3	10,8	1,0	± 1,9
PD1+ TH cells	tp 2	14,0	9,3	1,0	± 1,9

### LDRT Decreases the Numbers of Activated Immune Cells

As not only the absolute numbers of immune cells in the periphery are an indicator of a patient’s current immune status, activation markers on the immune cells were monitored in addition.

**Figure 5:**
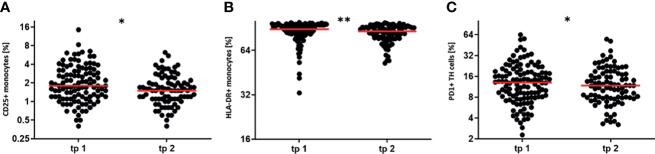
LDRT decreases the percentage of activated immune cells. The activation state of immune cells in the peripheral blood was determined by measuring the expression of distinct activation markers. Blood withdrawals were performed before (tp1) and after the serial irradiation (tp2). The expression levels of common activation markers were determined by flow cytometry. The Wilcoxon test was used for the statistical analysis. In the figure the percentage of CD25 positive and HLA-DR positive monocytes **(A, B)**, as well as the percentage of PD1 positive T Helper cells **(C)** is presented. (n = 125; *p < 0.05; **p < 0.01).

Indeed, not only modulations of cell numbers, but also a downregulation of different activated immune cell subtypes were found. **Figure 5** depicts those modulations.

A significant downregulation of the activation status was detected for the peripheral blood monocytes. These cells show a downregulation of CD25, which is part of the IL-2 cytokine receptor (**Figure 5A**). Moreover, also HLA-DR that is involved in antigen presentation, is downregulated on monocytes after the first series of irradiation (**Figure 5B**). A reduced active state was also determined for T Helper cells after irradiation. In detail, T Helper cells downregulated their expression of the activation marker PD1 (**Figure 5C**). The detailed parameters for the regulation of the activation markers are displayed in **Table 3**. In summary, these findings indicate that LDRT affects patient’s immune status by modulating absolute immune cell numbers (**Figure 4**), as well as the activation state of certain immune cell subtypes (**Figure 5**).

### Cells of the Monocytic Lineage Predict the Therapeutic Outcome

**Figure 6:**
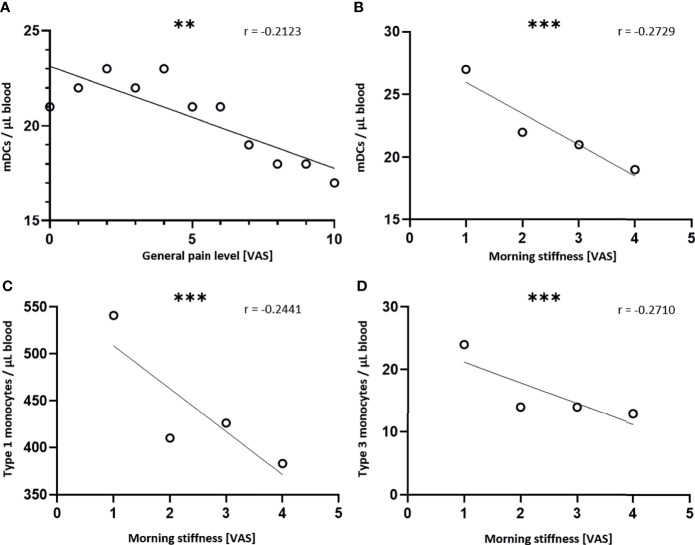
Cells of the monocytic lineage predict the therapeutic outcome. Absolute immune cell numbers were determined by flow cytometry before and after every serial irradiation, as well as for the follow-up appointment. At the same time points, the patients scored their subjective pain level in different situations on a VAS. A Spearman correlation was performed to assess the relationship between the immunological variables and the pain related variables. A simple linear regression was performed afterwards. For the graphical illustration, the mean values of the immune cell numbers were calculated and plotted. In **(A)**, the mean numbers of myeloid DCs (mDCs) are correlated to the general pain level and in **(B)** those numbers are correlated to the morning stiffness. In **(C)** the mean number of type 1 monocytes and in **(D)** the mean number of type 3 monocytes are correlated to the morning stiffness (n = 100; **p < 0.001; ***p < 0.001).

The data generated from the immunophenotyping is not only valuable to prove and unravel the underlying immunological effects of LDRT, but to define therapeutic and prognostic biomarkers that might predict the therapeutic outcome. Therefore, correlations of the pain level scores and the data of patient’s immune status were performed in order to identify immune biological factors that correlate with the therapeutic outcome. In fact, three immune cell subtypes correlated strongly to pain-related factors. All of these cell types belong to the monocytic lineage of blood leukocytes (**Figure 6** and **Table 4**).

**Table 4:** Statistical parameters for the modulation of the cells of the monocytic lineage.

Immune cell type	Pain level	Mean	SD	SEM	95% CI
mDCs	General Pain level 10	17	6	2,0	± 4,0
mDCs	General Pain level 9	18	6	1,8	± 3,6
mDCs	General Pain level 8	18	6	1,4	± 2,7
mDCs	General Pain level 7	19	6	1,1	± 2,2
mDCs	General Pain level 6	22	10	1,8	± 3,5
mDCs	General Pain level 5	22	10	1,8	± 3,6
mDCs	General Pain level 4	23	13	3,5	± 6,8
mDCs	General Pain level 3	22	8	1,9	± 3,7
mDCs	General Pain level 2	23	9	1,5	± 3,0
mDCs	General Pain level 1	22	6	1,6	± 3,2
mDCs	General Pain level 0	21	3	1,6	± 3,2
mDCs	Morning stiffness 4	19	7	0,7	± 1,5
mDCs	Morning stiffness 3	21	17	2,5	± 5,0
mDCs	Morning stiffness 2	22	9	1,5	± 2,9
mDCs	Morning stiffness 1	27	10	1,8	± 3,4
Type 1 monocytes	Morning stiffness 4	383	119	13,0	± 25,5
Type 1 monocytes	Morning stiffness 3	425	329	48,0	± 94,0
Type 1 monocytes	Morning stiffness 2	410	135	22,5	± 44,1
Type 1 monocytes	Morning stiffness 1	541	229	41,8	± 81,8
Type 3 monocytes	Morning stiffness 4	13	9	1,0	± 1,9
Type 3 monocytes	Morning stiffness 3	14	12	1,7	± 3,4
Type 3 monocytes	Morning stiffness 2	14	8	1,3	± 2,5
Type 3 monocytes	Morning stiffness 1	24	13	2,4	± 4,7

The numbers of mDCs show a significant negative correlation to the general pain level (r = -0.2123). This means that with increasing numbers of mDCs in peripheral blood, patients report a reduced pain level (**Figure 6A**). Additionally, the numbers of mDCs correlate negatively (r = -0.2729) to the morning stiffness, meaning that the morning stiffness is reduced when slightly higher levels of mDCs are found in the peripheral blood (**Figure 6B**). Not only dendritic cells predict the therapeutic outcome, but also type 1 monocytes (CD14high/CD16-), as well as type 3 monocytes (CD14low/CD16+) correlate negatively (r = -0.2441 and r = -0.2710, respectively) to the morning stiffness. This means that increasing morning stiffness comes along with reduced levels of monocytes. These findings might qualify cells of the monocytic lineage as potential biomarkers for predicting the therapeutic outcome of LDRT.

## Discussion

LDRT is well known for its pain-relieving effects in chronic degenerative and inflammatory diseases (25, 26). Indeed, the findings of the IMMO-LDRT01 trial strongly support this. In detail, the general pain level decreased during the course of the diseases, as well as the pain under load. Especially the reduction of pain under load is valuable for the patients, as thereby the mobility and the quality of life can be increased again. Morning stiffness is a common symptom in chronic degenerative diseases, especially in osteoarthritis and rheumatoid arthritis. Also, this symptom is significantly reduced in the patients after LDRT. As, there is still a significant pain reduction at tp 5 (three months after the last irradiation series), patients were asked to score their pain level again retrospectively at least 6 months after the last irradiation series. Even at this time point, patients still reported about a long-lasting pain reduction. As the majority of the patients was not responding sufficiently to previous therapies, this finding is even more impressive and supports the effectiveness of this therapy. For future studies these findings indicate that longer follow-up times are necessary to display the whole therapeutic effectiveness of LDRT (18).

Even though the pain-relieving effects of LDRT have already been well described in numerous patient studies, the underlying immunological mechanisms have to date not yet been analyzed in patient trials in a prospective manner. In the IMMO-LDRT01 trial, we show for the first time that LDRT has not only immunomodulatory effects in preclinical models and *in vitro* systems, but that it induces systemic modulations of immune cells of the peripheral blood in patients with chronic inflammatory and degenerative diseases. In detail, LDRT differentially modulates absolute cell numbers of circulating immune cells. Nonetheless, the numbers of the general leukocyte count remained stable after LDRT. This indicates that low dose radiation is not just affecting all circulating immune cells, rather than regulating specific immune cell types. Also, in the context of radiation protection, this fact is valuable, as it is indicating that low doses of X-Rays are not very harmful to healthy tissue.

For the immune cell subtypes, LDRT was found to upregulate the numbers of eosinophils and basophils, as well as the number of pDCs in peripheral blood. pDCs are connectors of the innate and the adaptive immune system as they activate cells of both branches of the immune system (27). Nonetheless, the function of pDCs is not only the activation of immune cells, but also the induction of immunological tolerance, especially T cell tolerance (28). Hence, an upregulation of those cells could be a sign of mitigated inflammatory processes. In line with that, we detected a significant downregulation of the numbers of circulating B cells. These cells of the adaptive immune system have a key role in antigen presentation and humoral immunity and therefore in the progression of immune responses (29). In addition, LDRT does not only regulate the absolute number of peripheral blood immune cells, but is affecting the activation state especially of monocytes and T Helper cells. After a first series of LDRT, monocytes reduced their expression of CD25, that is a common activation marker on leukocytes, as well as their expression of HLA-DR that is a key receptor for antigen presentation. Even though monocytes are mainly HLA-DR positive by definition (30), a downregulation of the expression is often characterized in immunosuppressive situations (31, 32). T cells are essential in mediating immune responses (33) and PD1 expression on T cells is a sign of previously being activated (34). A reduction of activated monocytes and T cells after LDRT is further supporting the hypothesis that LDRT is forming a rather anti-inflammatory milieu. The detected changes are minor but highly significant. This fact is remarkable as LDRT is delivered only locally to the affected joint but induces systemic immunomodulatory effects that can also be detected in peripheral blood. Since the here presented patient collective is rather heterogeneous, for example regarding the age or the indications of the patients, a certain heterogeneity in the immune status is expected. More prominent immunological modulations that are induced by LDRT are expected when patient subgroups are analyzed. Factors, such as gender, and age have a great impact on the immune status and might impact on the here presented data (35, 36). Additionally, all of the underlying indications are based on different biological and immunological mechanisms that might lead to changes in the peripheral blood immune status. In the explorative IMMO-LDRT01 trial we aimed to analyze for the first time peripheral blood immune alterations of patients that routinely do receive LDRT and the indications (displayed in **Table 1**) reflect the clinical routine. In the future, we will analyze patient subgroups defined by the respective disease. However, for this, more patients will have to be included. Additionally, we are currently preparing a prospective trial focusing on patients with arthritis.

The application of the immunophenotyping in clinical trials allows the longitudinal screening of numerous immunological parameters. These analyses are not only valuable to unravel the immunological modes of action of LDRT, but also to define predictive and prognostic biomarkers for LDRT. Therefore, we correlated pain related factors to the immunological data. Indeed, we found that cells of the monocytic lineage show a strong, negative correlation to the general pain level, as well as to the morning stiffness. The later symptom is often found in chronic degenerative and inflammatory diseases, particularly in osteoarthritis and rheumatoid arthritis (37, 38). As pain in arthritis and osteoarthritis is closely connected to the chronic inflammatory processes, a correlation of pain-related symptoms with immunological modulations is obvious (39). The cell numbers of mDCs correlate negatively to the general pain level and the morning stiffness. DCs represent a heterogeneous population of antigen presenting cells. Primarily, mDCs have a key role in initiating adaptive immune responses, but they are also key for tolerance induction and maintenance. Especially in chronic inflammation, that is a key feature in chronic degenerative and inflammatory diseases, mDCs can shift their phenotype towards a regulatory cell type. This shift is often triggered by signals delivered through anti-inflammatory cytokines, such as IL-10 and TGF-β (40). Just like mDCs, monocytes are as well a very heterogeneous cell population. Therefore, we determine three different monocytes subsets in the immunophenotyping panels. The classical monocytes (type 1 monocytes) that make up 90% of all blood monocytes, as well as the non-classical monocytes (type 3 monocytes) correlate negatively to the morning stiffness as well. Classical monocytes can differentiate into monocyte-derived DCs (moDCs), as well as macrophages, whereas non-classical monocytes are not likely to differentiate into moDCs (41). In general, monocytes can activate different branches of the immune system, such as TH1 or TH2 mediated immunity, but also bear tolerogenic functions by the activation of Treg cells that leads to an amelioration of pre-existing inflammatory processes. Which of these different mechanisms is initiated, is strongly depended on the immunological micro-milieu and cytokine signals (42). In order to analyze further how monocytes modulate the inflammation and pain sensation after LDRT, further functional analyses on the monocyte phenotype need to be performed in the future.

## Data Availability Statement

The raw data supporting the conclusions of this article will be made available by the authors, without undue reservation.

## Ethics Statement

The studies involving human participants were reviewed and approved by Institutional Review Board of the Friedrich-Alexander Universität Erlangen-Nürnberg (protocol code: 289_15B, date of approval: 12.11.2015). The patients/participants provided their written informed consent to participate in this study.

## Author Contributions

Conceptualization, UG, RF, OO, and BF. Methodology, A-JD, IB, TG, and TW. Software, A-JD, IB, BMF, and MD. Formal analysis, A-JD, BMF, MD, and LM. Investigation, A-JD and TW. Writing—original draft preparation, A-JD. Writing—review and editing, IB and UG. Visualization, A-JD. Supervision, UG and BF. Project administration, BF. Funding acquisition, UG and BF. All authors contributed to the article and approved the submitted version.

## Funding

This work is funded by the Bundesministerium für Bildung und Forschung (GREWIS and GREWIS-alpha, 02NUK017G und 02NUK050E).

## Conflict of Interest

The authors declare that the research was conducted in the absence of any commercial or financial relationships that could be construed as a potential conflict of interest.

The reviewer KL declared a past co-authorship with authors UG and BF to the handling editor.

## Publisher’s Note

All claims expressed in this article are solely those of the authors and do not necessarily represent those of their affiliated organizations, or those of the publisher, the editors and the reviewers. Any product that may be evaluated in this article, or claim that may be made by its manufacturer, is not guaranteed or endorsed by the publisher.
