# Corrigendum: Low Dose Radiation Therapy Induces Long-Lasting Reduction of Pain and Immune Modulations in the Peripheral Blood - Interim Analysis of the IMMO-LDRT01 Trial

**DOI:** 10.3389/fimmu.2022.859489

**Published:** 2022-02-07

**Authors:** Anna-Jasmina Donaubauer, Ina Becker, Thomas Weissmann, Birgitta M. Fröhlich, Luis E. Muñoz, Thomas Gryc, Manuel Denzler, Oliver J. Ott, Rainer Fietkau, Udo S. Gaipl, Benjamin Frey

**Affiliations:** ^1^ Translational Radiobiology, Department of Radiation Oncology, Universitätsklinikum Erlangen, Friedrich-Alexander-Universität Erlangen-Nürnberg (FAU), Erlangen, Germany; ^2^ Department of Radiation Oncology, Universitätsklinikum Erlangen, Friedrich-Alexander-Universität Erlangen-Nürnberg (FAU), Erlangen, Germany; ^3^ Department of Internal Medicine 3 -Rheumatology and Immunology, Friedrich-Alexander-University Erlangen-Nürnberg (FAU), Universitätsklinikum Erlangen, Erlangen, Germany

**Keywords:** low dose radiation therapy (LDRT), immune status, immunophenotyping, chronic degenerative and inflammatory diseases, subjective pain level, x-rays

In the original article, there was an error. The mistake is found in the sentence “While the total number of leukocytes remained unchanged in the peripheral blood, LDRT induced a slight reduction of eosinophils, basophils and plasmacytoid dendritic cells and an increase of B cells.” in the abstract of the article.

As it is correctly stated in the results section of the paper, the eosinophils, the basophils and the plasmacytoid dendritic cells increased, while the B cells were decreased.

A correction has been made to the **Abstract**:

“While the total number of leukocytes remained unchanged in the peripheral blood, LDRT induced a slight increase of eosinophils, basophils and plasmacytoid dendritic cells and a decrease of B cells.”

The authors apologize for this error and state that this does not change the scientific conclusions of the article in any way. The original article has been updated.

## Publisher’s Note

All claims expressed in this article are solely those of the authors and do not necessarily represent those of their affiliated organizations, or those of the publisher, the editors and the reviewers. Any product that may be evaluated in this article, or claim that may be made by its manufacturer, is not guaranteed or endorsed by the publisher.

